# The Rise of China in Fungal Taxonomic Studies and Biodiversity Cataloging in the Past Decade

**DOI:** 10.3390/jof12020101

**Published:** 2026-01-31

**Authors:** Ke Wang, Ming-Jun Zhao, Lei Cai

**Affiliations:** Fungarium, State Key Laboratory of Microbial Diversity and Innovative Utilization, Institute of Microbiology, Chinese Academy of Sciences, Beijing 100101, China; wangk@im.ac.cn (K.W.); zhaomj@im.ac.cn (M.-J.Z.)

**Keywords:** novel fungal taxa, Chinese scholars, national checklist of fungi, fungal data, new fungal species in China

## Abstract

Mycological studies in China have achieved substantial and encouraging progress in recent decades. In this paper, the discoveries of novel fungal taxa published by Chinese mycologists and the documented records of fungi distributed in China are statistically summarized based on the data retrieved from Fungal Names and the Checklist of Fungi in the China database, respectively. Our analysis reveals that a total of 2875 Chinese scholars have published 20,826 new fungal taxa to date, 65% of which were published within the past decade. During the same period, Chinese mycologists placed great importance on archiving fungal diversity data and have completed the compilation of the national checklist of fungi. Based on 382,503 records derived from over 18,200 research articles and 300 books, a total of 31,180 fungal species, spanning 17 phyla, 65 classes, 240 orders, 840 families, and 4531 genera, have been documented in China. The southwestern region, especially Yunnan Province, exhibits the highest richness of documented species. These results provide a comprehensive overview of the current status of fungal biodiversity and taxonomic studies in China, providing a valuable foundation for future investigations.

## 1. Introduction

The history of fungi-related knowledge and practice in China spans millennia, marked by early documentation, scholarly compilation, and contemporary scientific advancement. Fungi were first recorded in China as early as the second century during the Han Dynasty, with fourteen fungal species, including six species of *Ganoderma*, *Wolfiporia hoelen*, *Polyporus umbellatus*, etc., cataloged among 365 medicinal herbs in the ancient pharmacopeia Divine Farmer’s Classic of Materia Medica [1]. Over a millennium later, in 1245 DC, the first Chinese treatise on fungi was compiled, detailing the habitat, morphology, taste, and toxicity of eleven distinct species [2]. The modern scientific study of fungi in China was initiated by European scholars. The widely distributed stinkhorn mushroom *Lysurus mokusin* (L.f.) Fr. holds the distinction of being the first fungal species recorded from China, described by the French missionary Cibot in 1774 [3,4]. It was not until the early twentieth century that pioneering Chinese mycologists Fang-Lan Dai and Shu-Qun Deng initiated independent research in the field [5]. Following over a century of dedicated development, mycology in China has attained a position of notable achievement and international recognition [6].

In this study, data pertaining to fungal diversity in China were accessed from open-access databases to evaluate the current status of taxonomic and biodiversity research, with particular emphasis on advances made within the past decade.

## 2. Materials and Methods

The data on nomenclatural novelties for fungi were retrieved from the Fungal Names repository [7] (https://nmdc.cn/fungalnames/, accessed on 10 September 2025). The full datasets of 594,100 records of published fungal names were collected. Each record includes standardized metadata fields on scientific name, author(s), year of publication, journal, typification, type locality, host or substrate, synonyms, nomenclature comments, classification, name type, and name status. Of all the data, 18,153 records marked as ‘deleted’ in the Name Status column were first removed. When screening by ‘Fungi’ in the Classification column and ‘new taxon’ in the Name Type column, 179,892 records were excluded. We also excluded the invalid and illegitimate names. After data identification and screening, the final datasets of 355,007 records of published fungal names were collected and using in the analysis of Section 3.1.

The data on fungal diversity in China were retrieved from the Checklist of Fungi in the China database (https://nmdc.cn/fungarium/fungi/chinadirectories, accessed on 28 November 2025). The full datasets of 399,001 records of fungi reported in China were collected. Each record includes standardized metadata fields on scientific name, author(s), year of publication, journal, host or substrate, nomenclature comments, and classification. We excluded 15,915 records of out of the kingdom fungi and 583 records of invalid or illegitimate names. The final datasets of 382,503 records of fungi reported in China were collected and using in the analysis of Section 3.2.

**Figure 1:**
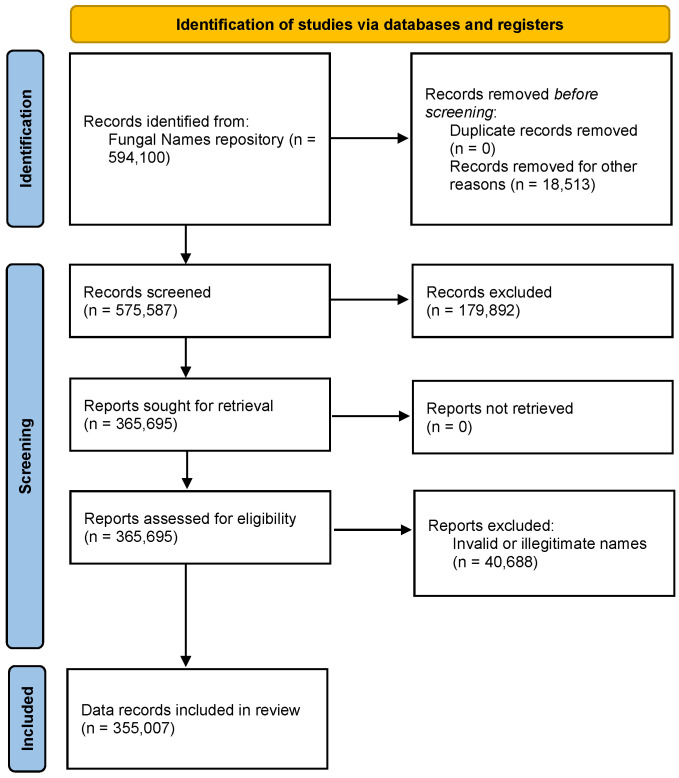
PRISMA flowchart of data from Fungal Names repository.

**Figure 2:**
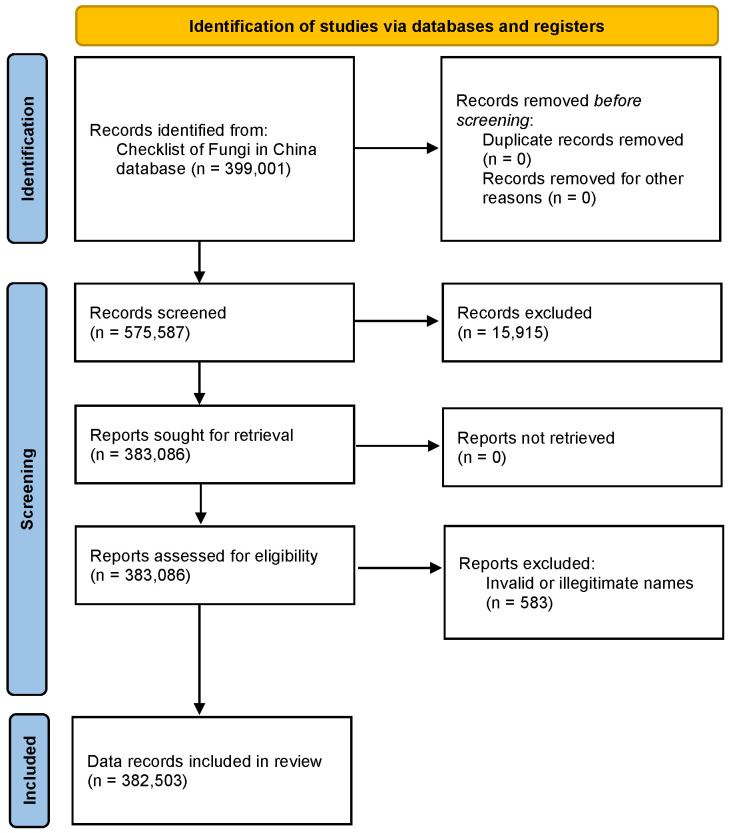
PRISMA flowchart of data from Checklist of Fungi in China database.

The review was conducted in accordance with PRISMA 2020 guidelines (Supplementary files). All authors participated in the initial data curation process and screened the data for relevance. Figure 1 and Figure 2 illustrate the steps of data processing from each database.

## 3. Results

### 3.1. Fungal Taxonomic Studies in China

#### 3.1.1. New Taxa Published by Chinese Scholars

**Figure 3:**
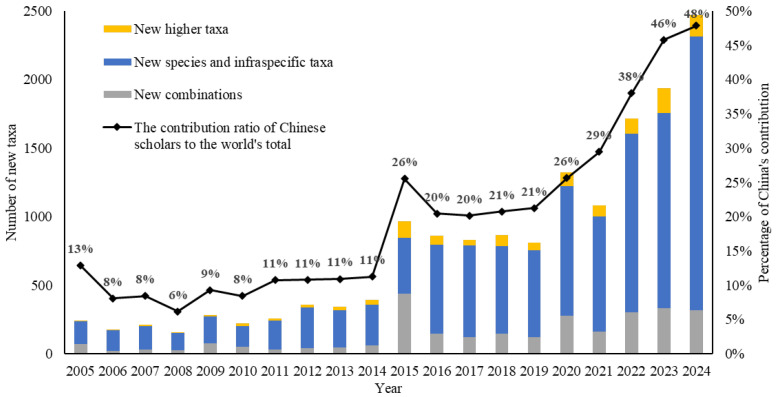
New fungal taxa published by Chinese authors and their contribution ration to the world’s total (2005–2024).

Since the publication of the fungal species *Uncinula nankinensis* F.L. Tai by Chinese mycologist Fang-Lan Dai in 1930, Chinese researchers have consistently endeavored to fill the gaps in our understanding of fungal diversity over the past century [5]. To date, Chinese scholars have collectively published 20,826 new fungal taxa, including 3 classes, 33 orders, 2 suborders, 168 families, 9 subfamilies, 1035 genera, 68 subgenera, 15,151 species, 328 infraspecific taxa, and 4029 combinations. Notably, 65% of these new taxa were published in the past decade. Starting from 2015, the annual number of novel fungal taxa reported by Chinese authors has increased by 1000, peaking at 1988 in 2024 [8]. Furthermore, China’s contribution to the world’s total has risen continuously, reaching a remarkable 48% in 2024 (Figure 3).

A total of 2875 Chinese scholars have contributed to the publication of new fungal taxa, accounting for 11.86% of the authors worldwide. Among them, 75 scholars have each published over 100 new fungal taxa. Leading contributors such as Yu-Cheng Dai, Lei Cai, Zhu-Liang Yang, Feng-Yan Bai, and Wen-Ying Zhuang all have long-standing research backgrounds in taxonomy and have made substantial contributions to the description and documentation of fungal diversity both in China and worldwide. Additionally, 1208 Chinese scholars (42.02%) have thus far published only a single new fungal taxon, constituting a notable proportion of “one-time” authors in the field.

#### 3.1.2. Favored Fungal Groups of Chinese Mycologists

Chinese mycologists have engaged in extensive research across all major fungal groups, including mushrooms, molds, yeasts, lichens, oomycetes, slime molds, and fossil fungi. Early investigations were largely centered on economically important plant pathogenic fungi, particularly those in the families Mycosphaerellaceae, Pucciniaceae, and Didymellaceae, an emphasis that continues to the present. In the past decade, however, studies on macrofungi, particularly wood-inhabiting fungi and agarics, have gained prominence and now represent a major focus of study (Table 1). During this period, Chinese mycologists have taken a leading role in reconstructing the phylogenetic framework for numerous important fungal groups, i.e., Basidiomycota [9,10], yeasts [11,12], agarics [13,14], polypores [15,16,17], slime molds [18], lichens [19,20], and plant pathogenic fungi [21,22]. Leveraging extensive specimen collections from China and worldwide, these contributions have not only led to the discovery of numerous new species but have also supported the establishment of higher rank classifications and the revisions of existing taxa.

#### 3.1.3. New Fungal Species from China

**Figure 4:**
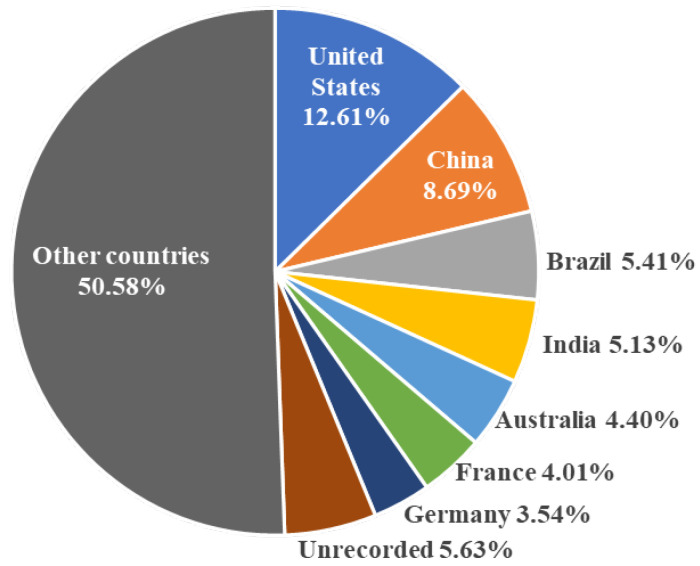
Contribution percentages of leading countries in the discovery of new fungal species.

A total of 15,266 new fungal species have been described from China, accounting for 8.69% of globally described species, placing the country second only to the United States (22,164 species, 12.61%; Figure 4). These new species have been reported from all 34 provincial-level regions of China, with Yunnan Province recording the highest count (3869 species), accounting for nearly one-fifth of the national total. Southwestern China exhibits the greatest richness of new fungal species. This region, characterized by mountainous terrain, high vegetation cover, a temperate climate, and abundant rainfall, is recognized as one of the world’s 34 biodiversity hotspots and provides an ideal environment for fungal growth and proliferation [23].

**Figure 5:**
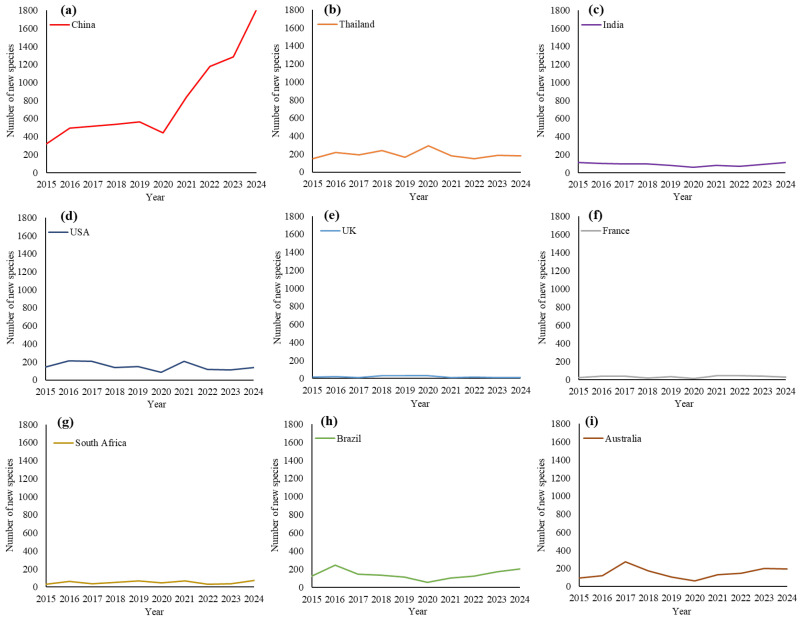
Annual new species discoveries of selected countries (2015–2024): (**a**) China, (**b**) Thailand, (**c**) India, (**d**) USA, (**e**) UK, (**f**) France, (**g**) South Africa, (**h**) Brazil, and (**i**) Australia.

A comparative analysis of leading countries in fungal taxonomic study, selected based on their current prominence or historical standing in the field, shows clear trends in the discovery of new species over the past decade (Figure 5). China emerges as the leading contributor to fungal taxonomy, exhibiting a sharply rising trajectory, with nearly 800 new species discovered per year. In contrast, other countries have maintained relatively stable levels, each recently reporting fewer than 200 new species annually.

### 3.2. The Fungal Diversity Catalogue in China

#### 3.2.1. The Checklist of Fungi in China

**Figure 6:**
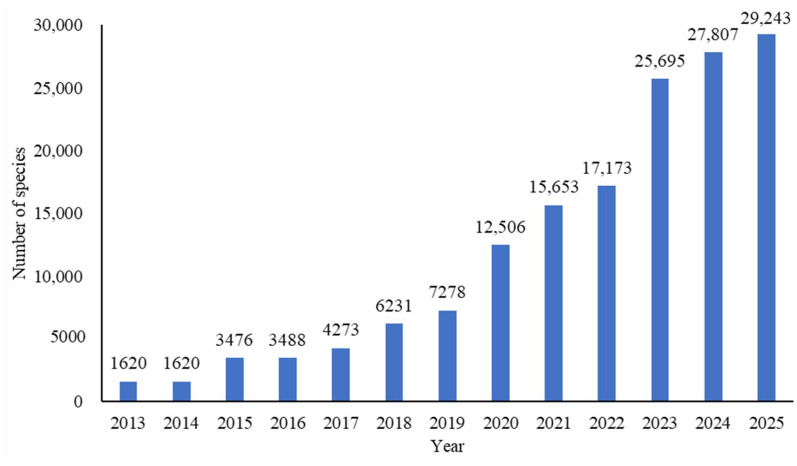
Increase in fungal records in the Catalogue of Life China in recent years.

Biodiversity cataloging initiatives are increasingly recognized for their importance at both global and regional levels, and China has made notable progress in this area. The Catalogue of Life China project, initiated in 2007 under the auspices of the Biodiversity Committee of the Chinese Academy of Sciences, involves domestic and international taxonomists across various organism groups documenting all known species present in China. In 2011, the fungi group is formally included in the project, leading to the establishment of the Checklist of Fungi in the China database, designed to collect fungal diversity data from digitalized historical documents. To date, this effort has integrated over 380,000 fungal records derived from approximately 18,200 research articles and more than 300 monographs. This resulting repository provides a comprehensive overview of the fungal diversity in China and serves as the essential foundation for compiling the national fungal checklist. The inaugural checklist, comprising 1670 species, was issued in 2013 (Figure 6). Over the following decade, hundreds to thousands of species were incorporated each year, culminating in the 2025 edition that documented 29,243 fungal species and infraspecific taxa [24]. The accompanying online database (http://www.sp2000.org.cn/) allows users to retrieve integrated information for each species, including current and synonymous names, common names, geographic distributions, economic value, and relevant references.

#### 3.2.2. The Known Fungal Diversity in China

Based on the Catalogue of Life China 2025 Annual Checklist [24] and incorporating the latest updates from the Checklist of Fungi in the China database, we have analyzed the current state of knowledge regarding fungal diversity in China. There have been 31,180 species documented in the country (to be issued in the Catalogue of Life China 2026 Annual Checklist). This diversity spans 17 phyla, 65 classes, 240 orders, 840 families, and 4531 genera, representing approximately 17.74% of the world’s known fungal diversity (Table 2).

**Figure 7:**
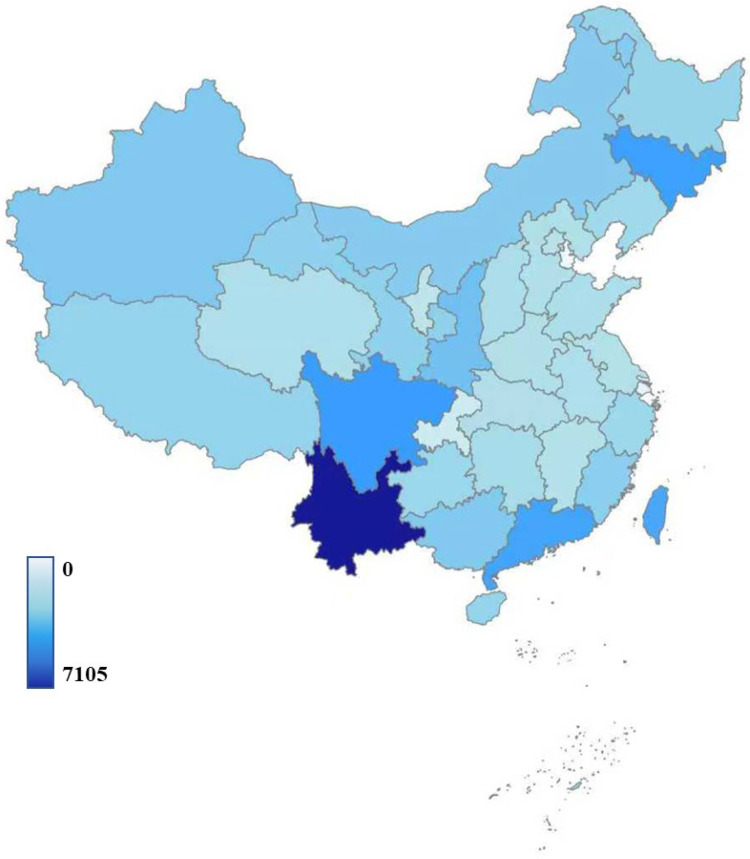
Distribution map of fungal species diversity in China.

Seventeen out of the nineteen known phyla of fungi have been reported in China, among which are Ascomycota and Basidiomycota that exhibit the richest diversity (Table 2). The most species-rich families are Mycosphaerellaceae and Pucciniaceae, each with over 1000 recorded species (Table 3). Geographically, southwest China exhibits the highest diversity (Figure 7). Notably, Yunnan alone has recorded 7105 fungal species, accounting for 22.79% of the national total. In addition to the southwest, several other regions of China also exhibit high fungal diversity. In southeastern Taiwan, southern Guangdong, and northeastern Jilin, each has recorded approximately 3000 or more fungal species. The rapid increase in known species in Xizang in recent years is also noteworthy [25,26,27], largely attributable to the Second National Investigation of the Qinghai–Xizang Plateau. China’s vast territory, together with substantial regional variations in climate and geography, has fostered rich and distinctive fungal biodiversity across many areas of the country.

#### 3.2.3. Red List of China’s Biodiversity—Macrofungi

**Figure 8:**
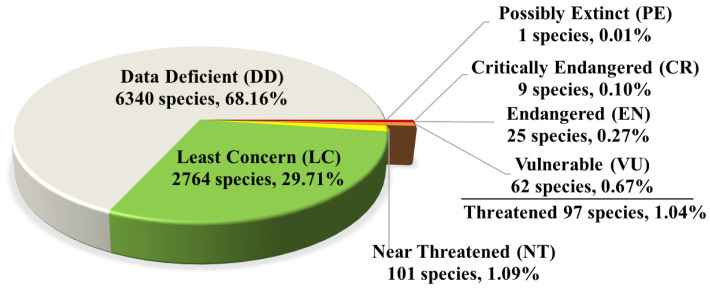
Categories of assessment of macrofungi in the Red List [32].

Environmental pollution, climate change, over-exploitation, and other anthropogenic factors have posed serious threats to the survival and biodiversity of macrofungi worldwide [28]. In 2018, the National Red List of Fungi for China was officially published jointly by the Ministry of Ecology and Environment of China and the Chinese Academy of Sciences [29]. The assessment covered 9302 species of macrofungi distributed in China, including 870 ascomycetes, 6268 basidiomycetes, and 2164 lichens. Among these, 97 species were assessed as threatened: one Possibly Extinct (PE), nine Critically Endangered (CR), 25 Endangered (EN), and 62 Vulnerable (VU). The remaining categories included 101 Near Threatened (NT), 2764 Least Concern (LC), and 6340 Data Deficient (DD). No species were assessed as Extinct (EX) or Wild Extinct (EW) (Figure 8).

The only species classified as Possibly Extinct is *Hemiglossum yunnanense*. First described in 1890 based on a single specimen collected in Yunnan [30], it has not been rediscovered despite repeated surveys of the type locality [31]. As the presence of viable spores in the soil remains uncertain, it was assessed as Possibly Extinct rather than Extinct. Several economically significant edible and medicinal fungi were also assessed as threatened. *Ophiocordyceps sinensis*, one of the world’s most renowned medicinal fungi, was assessed as Vulnerable. Its natural populations have declined sharply in recent decades as a result of climate change and overharvesting [31,32]. Widely consumed edible mushrooms such as *Hericium erinaceus* and *Hypsizygus marmoreus* were also considered as threatened. Although these species are now cultivated artificially, their wild populations continue to decline due to habitat loss [32].

#### 3.2.4. Flora Fungorum Sinicorum

To achieve a comprehensive understanding and promote the sustainable utilization of fungal resources, the compilation of the ‘Flora Fungorum Sinicorum’ was initiated in 1973. Since the publication of its inaugural volume in 1987, this long-standing project has spanned half a century, embodying the sustained dedication of successive generations of mycologists [33,34,35,36]. To date, 74 volumes have been published, comprising over 30 million words and documenting more than 1000 genera and 10,500 species and subspecific units of fungi recorded in China. Distinct from a checklist, Flora Fungorum Sinicorum offers comprehensive entries for each species, including detailed morphological and ecological descriptions, geographical distribution, and notes on human uses and potential hazards. It serves as both an authoritative reference and a comprehensive resource, supporting research, education, and application in China. Most importantly, within China’s highly competitive scientific funding system, the ‘Flora Fungorum Sinicorum’ has played an indispensable role in sustaining the country’s fungal taxonomy research teams.

## 4. Discussion

**Figure 9:**
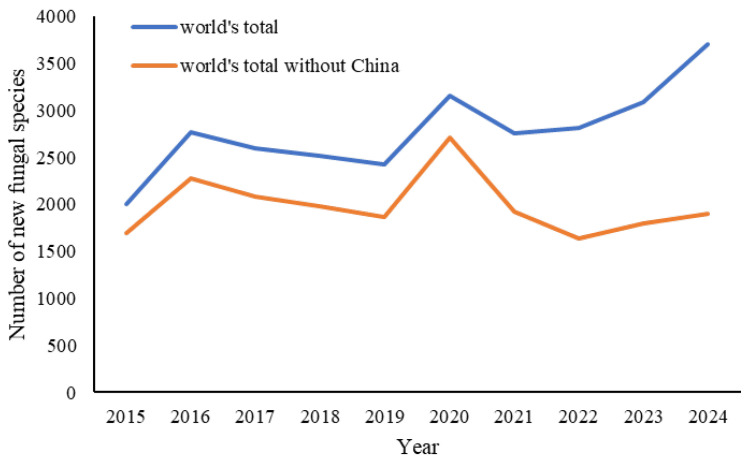
Annual new fungal species discoveries of the world (2015–2024).

Since the last century, concerns over the decline in taxonomy have been repeatedly raised [37,38,39,40,41], yet few of these discussions have incorporated data specific to fungal taxonomy. A recent study based on historical records of published fungal names indicated that the numbers of published fungal names and taxonomists increased sharply in the late 20th century, a growth trend that has accelerated in the 21st century [7]. Nevertheless, as shown in Figure 3, this upward trend fails to reflect the uneven development of fungal taxonomy across different countries and regions. The gradual increase in China’s contribution to global taxonomic output may have skewed the overall assessment of the global landscape and the status of most other countries. When Chinese data are excluded, the annual number of newly described fungal species worldwide has remained relatively stable over the past decade, and even showed a slight downward trend after peaking in 2020 (Figure 9). Although it is still too early to conclude that fungal taxonomy is in decline, the long-term prospects of the field warrant sustained attention. Without such vigilance, achieving a comprehensive understanding of global fungal diversity will remain out of reach in the foreseeable future.

Despite significant advances of mycological study in China, a comprehensive understanding of its fungal diversity, and its subsequent sustainable utilization and effective conservation, remains a long-term endeavor. The global number of fungal species is currently estimated to range from 2 to 3 million with a “best estimate” at 2.5 million [42]. Applying a commonly used estimation approach, specifically a fungus-to-plant ratio of 9.8:1 [43] and the more than 40,000 known vascular plant species in China [24], the estimated fungal diversity in China reaches approximately 400,000 species. According to the annual review of fungal nomenclatural novelties [8], taxonomic and biodiversity research efforts in China have generally stabilized, with over 1500 new species and new records of fungi being discovered annually. Even at this pace, fully documenting the country’s full fungal diversity would take nearly 250 years. Currently, the total holdings of fungal specimens in China amount to only 1.2 million (accessed from Furgarium Union of China, https://nmdc.cn/fuc/), a number smaller than the collections of the Royal Botanical Garden, Kew (UK), a single major international herbarium. Given China’s vast amount of land area, many regions remain understudied from a mycological perspective. As shown in the Red List of China’s Biodiversity—Macrofungi, 68.16% of the assessed macrofungal species are listed as Data Deficient (Figure 8), which starkly underlines the knowledge gaps regarding fungal diversity and conservation across the country. Moreover, numerous Chinese specimens, previously identified solely based on morphologically defined circumstance, have been re-assessed using phylogenetic and genomic approaches; many have been confirmed to represent distinct species, with a significant proportion identified as new species endemic to China or East Asia [44,45,46]. Therefore, in addition to continued exploration of new and fresh specimens, existing historical collections also warrant critical reexamination [47]. Establishing a comprehensive understanding of fungal diversity in China, and globally, remains a substantial and continuing scientific challenge.

## 5. Conclusions

This review systematically summarizes the remarkable achievements of Chinese mycologists in fungal taxonomic studies and biodiversity cataloging over the past decade, highlighting China’s rising status in global mycology. To date, Chinese scholars have described 20,826 new fungal taxa, with 65% of these discoveries made in the last ten years, and China’s contribution to global new fungal species reached an impressive 48% in 2024. The compilation of the Checklist of Fungi in China has systematically documented 31,180 fungal species in China, which provides a crucial foundation for regional fungal biodiversity research. Despite China’s notable achievements, the uneven development of fungal taxonomy across different countries could have emerged as a global concern. Current constraints in research efforts and funding further underscore the need to improve the efficiency of taxonomic and biodiversity studies. DNA data are transforming fungal classification at all levels [42]. Emerging technologies such as artificial intelligence (AI) offer promising potential to advance taxonomy by enabling the rapid recognition of known taxa and uncovering cryptic diversity [48]. Regardless of technological advancement, the role of taxonomists remains indispensable for their in-depth understanding and unique perspective of species traits. Overall, given that over 90% of fungal species are still undiscovered, fungal taxonomy must sustain and further enhance its vitality over a considerable time in the future.

## Figures and Tables

**Table 1:** Top 10 taxonomic groups studied by Chinese scholars in history and recent decade.

Taxonomic Group	Number of New Taxa Published Historically	Taxonomic Group	Number of New Taxa Published in the Last Decade
Mycosphaerellaceae	780	Boletaceae	365
Hymenochaetaceae	562	Hymenochaetaceae	354
Boletaceae	556	Polyporaceae	295
Polyporaceae	520	Tubeufiaceae	261
Xylariaceae	403	Agaricaceae	257
Agaricaceae	324	Didymellaceae	255
Pucciniaceae	313	Chaetosphaeriaceae	240
Aspergillaceae	296	Xylariaceae	227
Tubeufiaceae	290	Chaetomiaceae	217
Didymellaceae	289	Russulaceae	209

**Table 2:** The number of known species for major fungal groups in China and worldwide.

Phylum	Known Species in China	Known Species Worldwide	Proportion of China to the World
Aphelidiomycota	0	12	0.00%
Ascomycota	18,570	112,817	16.46%
Basidiobolomycota	4	10	40.00%
Basidiomycota	11,830	59,295	19.95%
Blastocladiomycota	21	204	10.29%
Calcarisporiellomycota	1	2	50.00%
Chytridiomycota	41	993	4.13%
Entomophthoromycota	130	308	42.21%
Entorrhizomycota	4	21	19.05%
Glomeromycota	184	350	52.57%
Kickxellomycota	41	353	11.61%
Monoblepharomycota	4	37	10.81%
Mortierellomycota	38	154	24.68%
Mucoromycota	245	764	32.07%
Neocallimastigomycota	7	46	15.22%
Olpidiomycota	12	64	18.75%
Rozellomycota	9	85	10.59%
Sanchytriomycota	0	2	0.00%
Zoopagomycota	39	233	16.74%
Total	31,180	175,750	17.74%

**Table 3:** Top 10 genera and families with the highest number of recorded species.

Family	Known Species in China	Genus	Known Species in China
Mycosphaerellaceae	1344	*Puccinia*	717
Pucciniaceae	1092	*Pseudocercospora*	463
Boletaceae	716	*Cercospora*	350
Polyporaceae	610	*Russula*	308
Russulaceae	592	*Meliola*	307
Parmeliaceae	538	*Trichoderma*	286
Hymenochaetaceae	491	*Colletotrichum*	257
Pleosporaceae	448	*Phyllosticta*	251
Meliolaceae	448	*Amanita*	242
Nectriaceae	446	*Alternaria*	238

## Data Availability

The data presented in this study are openly available in the Fungal Names repository (https://nmdc.cn/fungalnames/, accessed on 10 September 2025) and the Checklist of Fungi in China databases (https://nmdc.cn/fungarium/fungi/chinadirectories, accessed on 28 November 2025).

## Supplementary Materials

The following supporting information can be downloaded at: https://www.mdpi.com/article/10.3390/jof12020101/s1, File S1: PRISMA 2020 checklist; File S2: PRISMA 2020 abstract checklist.
